# Implementation of a smoke-free policy in a high secure mental health inpatient facility: staff survey to describe experience and attitudes

**DOI:** 10.1186/1471-2458-13-315

**Published:** 2013-04-08

**Authors:** Angela M Hehir, Devon Indig, Shani Prosser, Vicki A Archer

**Affiliations:** 1 Centre for Health Research in Criminal Justice, Justice and Forensic Mental Health Network (J&FMHN), Suite 302, Level 2, 152 Bunnerong Rd, Eastgardens, NSW, 2036, Australia; 2 School of Public Health and Community Medicine, University of New South Wales, Sydney, NSW, 2052, Australia; 3 J&FMHN, 1300 Anzac Parade, Malabar, NSW, 2036, Australia; 4 J&FMHN, Suite 302, Level 2, 152 Bunnerong Rd, Eastgardens, NSW, 2036, Australia

**Keywords:** Smoke-free policy, Psychiatric hospital, Staff, Attitudes, Smoking

## Abstract

**Background:**

In 2008, a new forensic hospital was opened as a totally smoke-free facility. This study describes the attitudes and experience of mental health professionals working in the high secure mental health facility three years after it was opened. It is part of a larger evaluation describing the experience of current and discharged hospital patients.

**Methods:**

Quantitative data was collected using a survey of hospital staff (N = 111) with a 50% response rate. The survey collected demographic and smoking data to describe staff responses to statements relating to hospital smoking policy, patient care and staff support.

**Results:**

Among staff surveyed, 13% were current smokers and 41% were ex-smokers (10% quit after commencing employment in the smoke-free hospital). Most (88%) preferred to work in a smoke-free environment, although this was significantly lower in smokers compared to non-smokers (39% vs. 95%). While most staff felt that the smoke-free environment had a positive impact on the health of patients (86%) and on themselves (79%), smokers were significantly less likely to agree. Just over half (57%) of staff surveyed agreed that patient care was easier in a totally smoke-free environment, although less smokers agreed compared to non-smokers. Staff who smoked were also significantly less likely to indicate they had sufficient support working in a smoke-free environment, compared to non-smokers (15% vs. 38%).

**Conclusions:**

The staff surveyed supported the smoke-free workplace policy; most agreed that patient care was easier and that the policy did not lead to an increase in patient aggression. Implementation of a total smoking ban can result in positive health outcomes for patients and staff, and may influence some staff to quit. Staff who smoke have a less positive experience of the policy and require additional support.

## Background

The rate of smoking in Australia among persons aged 14 years and over was 15.1% in 2010 [1]. Although rates have declined among the general population, the decline has not been observed in a number of disadvantaged groups including people with mental illness. Studies have observed rates of smoking up to three times higher among those with mental illness compared to general population rates (even higher rates being observed among patients with schizophrenia), with heavier smoking and lower rates of cessation also observed [2,3]. The highest levels of smoking have been observed in psychiatric inpatient units where rates of smoking have been estimated to be 70%, with 50% of smokers smoking heavily [4]. Smoking cessation is often not managed routinely and effectively within mental health settings due to limited staff training, communication difficulties and the low priority given to cessation. This is despite morbidity and mortality related to high rates of tobacco use exceeding other drug effects at a population level [5,6].

Many mental health inpatient settings in Australia and internationally have implemented or are in the process of implementing partial or total smoking bans [7-9]. Smoke-free policy implementation in mental health inpatient settings comes as a result of a wider move to ban smoking in hospitals and health services to reduce risks to staff and patients from environmental tobacco smoke [8]. Mental health facilities have been slower than other parts of the health service to implement total bans due to concerns including anticipated increases in aggression, symptom and behaviour management and patient rights issues [8,10-12].

Where policies have been effectively implemented in psychiatric inpatient facilities, key features include staff education and support, patient preparation, provision of nicotine replacement therapy (NRT) and an overarching strategy aimed at long term quitting incorporating evidence-based treatment and change in the smoking culture [8,13,14]. The role of staff has been identified as critical to the successful implementation of smoking bans in inpatient mental health facilities [7,11,15,16]. A survey of clinical and non-clinical staff in a large NSW psychiatric hospital conducted before the implementation of a total smoking ban revealed that while most staff thought the ban would improve their work environment, help staff stop smoking and improve the physical health of patients, they perceived barriers including the fear of patient aggression and likely patient non compliance [17]. The perception of increased aggression is despite the growing literature on smoking bans in mental health inpatient facilities which does not provide evidence for increases in aggression or violence by patients post ban [8,10,18-20]. The loss of cigarettes as a patient management tool within psychiatric inpatient settings has been presented as another barrier to change [11,12,15]. A recent survey of Nurse Managers in Australian mental health inpatient units found approximately one third believed it was useful to smoke with patients. The same survey also found that less than one fifth had received training related to the provision of nicotine dependence treatment [21]. Staff knowledge and attitudes to smoke-free policy have been identified as barriers to the provision of dependence support for mental health inpatients. Staff report concerns relating to the effects and costs of NRT, the loss of smoking as a patient coping strategy and the perception that patients are too unwell to quit while hospitalised [13]. The smoking status of staff has also been shown to impact on support for smoke-free policies, presenting additional obstacles to policy implementation [22-24]. Smoking status of health care providers in mental health settings has been demonstrated to impact on attitudes to smoke-free policy, engagement in interactions related to tobacco and motivation to provide cessation support to patients [25-27]. Staff beliefs can significantly impact on the implementation of smoke-free policies within mental health settings, indicating a need for organisational responses to the local work environment [26,27].

Forensic Mental Health is a specialised field, providing a pathway for forensic rehabilitation in an environment of high security. The hospital described in this paper provides specialist mental health care for individuals found not guilty of a crime by reason of mental illness or unfit to plead, those transferred from correctional or detention centres for mental health treatment and high risk civil patients. The hospital strives to provide optimum care of mentally ill patients while ensuring the safety of all patients, staff and the community

The study reported here describes the experience and attitudes of staff of the hospital, opened in 2008 in New South Wales, Australia as a totally smoke-free hospital. It is part of a larger evaluation which also includes the experience and attitudes of current and discharged patients. We report on the findings from the patient experience elsewhere [28]. The purpose of this paper is to describe the attitudes of staff and their experience of the total smoking ban. The differences observed between smoking and non-smoking staff will be described, along with implications for effective policy implementation.

## Methods

### Setting and participants

The facility, located in metropolitan Sydney NSW, provides high secure mental health inpatient care for up to 129 adult patients and a small number of adolescent patients. The hospital was opened in November 2008 as a totally non-smoking facility in compliance with New South Wales Health smoke-free workplace policy [29]. Under this policy, patients and staff are unable to smoke inside buildings or on the grounds of the hospital. At the time of the evaluation, there were 222 staff employed at the hospital including nursing (58%), medical (9%), allied health (10%), management (clinical and administrative) (19%) and administrative staff (6%). Hospital staff include those transferred from the adjacent correctional centre where smoking was allowed in designated outdoor smoking areas as well as newly recruited staff. Full and part time staff were eligible to participate in the evaluation. Contract staff were not included in the study. Ethics approval was obtained from the Justice Health Human Research and Ethics Committee.

### Provision of smoking cessation support

Prior to admission, forensic and correctional patients are assessed in the correctional centre’s health centre for nicotine dependence and offered nicotine replacement therapy (NRT) to assist them to cut down or quit. NRT offered includes patch, lozenge or inhaler. On admission, patients are assessed and withdrawal monitored.

During the evaluation period the hospital had no provisions for patients to access outside leave. Opportunities for staff to leave the hospital during working hours were limited, involving security screening on leaving and returning to the hospital.

### Procedure

The staff survey used a cross sectional survey design. The self report questionnaire was made available to all staff in the hospital employed by the health service. Surveys were developed based on the aims of the research and previously published work [12,17]. A focus group and interviews were also conducted with a sample of nursing and medical staff and analysed to inform survey development. Surveys were pilot tested with health care professionals from other parts of the health service and with Clinical Nurse Consultants within the hospital who did not participate in the survey. Participants in the pilot testing were representative of staff to be included in the survey and included smokers and non smokers and staff who provide care to patients with mental illness. The survey was reviewed for appropriateness of wording, clarity of both content and instructions and to ensure items elicited intended responses. The three page survey consisted of 27 questions. The first section focused on demographic information and smoking status. The second section consisted of a series of statements and a Likert scale to ascertain staff attitudes to aspects of the smoke-free policy. All questions were tick box, but staff did have the opportunity to write comments. The surveys were distributed to staff in consultation with hospital management. Distribution methods included email, staff meeting attendance and provision of hard copies with a return envelope or a return box in staff stations.

### Analysis

Survey responses were analysed using IBM SPSS Statistics Version 19. The responses to the 5 point Likert scale were collapsed to 3: Strongly Agree/Agree; Unsure; Strongly Disagree/Disagree. Demographic data and responses to statements relating to the smoking policy were analysed using descriptive statistics. Pearson chi square (2-tailed) was used to detect any significant differences in responses to statements relating to smoking policy between smokers and non-smokers at the P < 0.05 level.

## Results

### Participants

Of a total staff of 222, 111 (50%) completed the survey. Participants were most likely to be female and within the 30–39 year age bracket (Table 1). The majority of respondents were nurses and had worked in the hospital for over two years. The gender and nominated profession of respondents reflected the distribution of staff employed at the time of the evaluation. Fifty four percent of all staff in the hospital at the time of the evaluation were female. At the time of the evaluation 58% of all staff were classified as nursing staff, 19% management (including clinical and administrative), 10% allied health, 9% medical and 5% administrative staff.

**Table 1:** Demographic characteristics of survey respondents (n = 111)

**Demographic details**	**Number of respondents (%)**
Gender	
Male	49 (44.1%)
Female	62 (55.9%)
**Age**	
<20 years	1 (0.9%)
20–29 years	16 (14.4%)
30–39 years	43 (38.7%)
40–49 years	20 (18%)
50–59 years	29 (26.1%)
>60 years	0
No response	2
**Professional group**	
Management – Administration	8 (7.2%)
Management - Clinical	18 (16.2%)
Nursing	59 (53.2%)
Medical Officer / Staff Specialist	8 (7.2%)
Allied Health	14 (12.6%)
Administration	4 (3.6%)
**Years working at the hospital**	
<1 year	17 (15.3%)
Between 1 and 2 years	32 (28.8%)
>2 years	60 (54.1%)
No response	2 (1.8%)

### Smoking status of respondents

Of the 111 survey respondents, fourteen indicated that they were current smokers (Table 2). Of participants who indicated that they had ever smoked, 11 indicated that they had quit since commencing work at the Forensic Hospital.

**Table 2:** Smoking characteristics of survey respondents (n = 111)

**Smoking status**	**Number of respondents (%)**
**Ever smoked cigarettes**	
Yes	60 (54.1%)
No	50 (45.0%)
No response	1 (0.9%)
**If quit, when quit**	
Prior to working in the hospital	33 (29.7%)
Since working in the hospital	11 (9.9%)
No Response	4 (3.6%)
**Currently smoking**	
Yes	14 (12.6%)
No	46 (41.4%)
No Response	1 (0.9%)

### Staff attitudes to smoke-free policy

Staff overwhelmingly responded that they preferred to work in a totally smoke-free environment (Table 3). Staff who smoked were significantly less likely to prefer to work in a smoke-free environment, compared to non-smokers. More than twice as many smokers as non-smokers had concerns about working in a smoke-free environment before working at the hospital, but this difference was not statistically significant. One third of respondents felt that patients should not be forced to stop smoking; this was significantly higher among smoking staff. Staff who smoked were significantly less likely to report that providing nicotine dependence treatment to patients was as important as their other roles in the unit. Over half of the respondents reported that nicotine withdrawal is a significant issue for most patients in the hospital. There were no significant differences between smoking and non-smoking staff regarding their confidence in providing advice and treatment to smokers to help them cope with not smoking.

**Table 3:** Survey respondents’ attitudes to the smoke-free policy – percentage agreement for non-smokers and smokers

**Statement**	**% Agreement (Strongly Agree/Agree)**			
	**All respondents**	**Non-smokers (N = 95)**	**Smokers (N = 14)**	**Significance – Pearson χ**^**2 **^**(2 tailed)**
I prefer to work in a smoke-free environment	88.1	94.6	38.5	χ^2^ = 34.09; df = 2; *P <* 0.001
I had concerns or worries about working in totally smoke-free environment before commencing work in the hospital	18.9	15.0	38.5	χ^2^ = 4.52; df = 2; *P* = 0.104
Mental health inpatients should not be forced to stop smoking	34.0	29.5	64.3	χ^2^ = 13.13; df = 2; *P* = 0.001
Providing nicotine dependence treatment to patients is as important as other roles in the unit	80.0	83.5	57.1	χ^2^ = 6.31; df = 2; *P* = 0.043
I am confident in my ability to provide advice and treatment to smokers to help them cope with not smoking	66.0	65.9	69.2	χ^2^ = 1.37; df = 2; *P* = 0.504
Nicotine withdrawal is a significant issue for most patients in the hospital	57.0	58.2	57.1	χ^2^ = 5.28; df = 2; *P* = 0.071

### Staff experience of policy implementation and impact

Just over a third of respondents indicated that they believed most patients were prepared for smoking cessation prior to their admission to the smoke-free hospital (Table 4). While over half of all staff believed patient care was easier, this was lower in smokers than non-smokers. Smokers were also more likely to agree that patients were more aggressive and more difficult to manage under the policy, although the overall agreement with this statement was low. Staff who smoked were more pessimistic about the likelihood that patients would succeed at smoking cessation in the long-term compared to non-smoking staff, although this difference was not significant. Staff overwhelmingly agreed that the smoke-free environment had a positive effect on patients’ health, although smokers were less likely to agree than non-smokers. Most respondents agreed that the policy had a positive impact on their own health, but this was significantly lower in smokers compared to non-smokers.

**Table 4:** Respondents’ views on policy implementation and impact – percentage agreement for non-smokers and smokers

**Statement**	**% Agreement (Strongly Agree/Agree)**			
	**All respondents**	**Non-smokers**	**Smokers**	**Significance – Pearson χ**^**2 **^**(2 tailed)**
Most patients have been prepared for smoking cessation before they arrive at the hospital	38.1	40.4	28.6	χ^2^ = 3.84; df = 2; *P* = 0.146
Being in a totally smoke-free environment makes patient care easier	57.0	63.7	21.4	χ^2^ = 9.36; df = 2; *P* = 0.009
The smoke-free policy has made patient behaviour more difficult to manage	23.8	22.2	30.8	χ^2^ = 4.96; df = 2; *P* = 0.084
Mental health patients who are not allowed to smoke become more aggressive and hard to manage	19.8	16.5	38.5	χ^2^ = 8.29; df = 2; *P* = 0.016
Mental health patients who smoke are unlikely to ever quit long term	40.6	37.4	61.5	χ^2^ = 2.79; df = 2; *P* = 0.247
Living in the smoke-free hospital has had a positive effect on the health of patients.	85.5	88.6	61.5	χ^2^ = 6.87; df = 2; *P* = 0.032
Working in a smoke-free environment has had a positive impact on my health	79.0	86.8	23.1	χ^2^ = 29.37; df = 2; p < 0.001

### Support for staff who smoke

Just over half of all staff believed that it was difficult for staff who smoke to adhere to the hospital’s smoke-free policy (Table 5); there was no significant difference between smokers and non-smokers. About a third of respondents believed that staff who smoke receive adequate support from hospital management, with fewer smoking staff than non-smoking staff agreeing with the statement.

**Table 5:** Respondents’ views on experience of policy for smoking staff – percentage agreement for non-smokers and smokers

**Statement**	**% Agreement (Strongly Agree/Agree)**			
	**All respondents**	**Non-smokers**	**Smokers**	**Significance - Pearson χ**^**2 **^**(2 tailed)**
It is difficult for staff who smoke to adhere to the hospital’s smoke-free policy.	56.7	56.2	69.2	χ^2^ = 2.08; df = 2; *P* = 0.353
Staff who smoke receive adequate support from the hospital to enable them to work in the smoke-free environment.	35.0	37.5	15.4	χ^2^ = 11.97; df = 2; *P* = 0.003

## Discussion

The research reported here highlights the value mental health professionals place on working in a smoke-free environment, as well as confirming that most staff do not experience negative outcomes in terms of patient care or behaviour. The smoking rate among staff was slightly lower than the Australian rate of 15.1% for adults over 14 years of age [1], and the rate described for mental health inpatient staff reported in similar studies [17,28-30]. Among staff who had quit, a small number had quit since commencing work at the smoke-free facility. Implementation of smoke-free workplace policies have demonstrated reductions in staff smoking rates particularly when they incorporate a comprehensive approach including staff education and support [31]. The results observed in this study may reflect this reduction, although there may also be an effect of self selection among staff applying to work at the smoke-free hospital, resulting in smaller numbers of smokers. Restricting smoking among staff and treatment of nicotine dependence are important aspects of effective policy implementation [32]. Tobacco use by staff can act as a barrier to implementing smoke-free policies and supporting patients to quit [5,26,27]. A totally smoke-free workplace provides additional incentives to quit by limiting opportunities to smoke and creating a non-smoking culture within the setting.

Only one third of staff felt that smoking staff received adequate support from the hospital. This response came from both smokers and non-smokers, but was significantly lower among staff who smoked. Bloor et al. [31] evaluated the impact of a non-smoking policy on the smoking behaviour of mental health nurses and their attitudes to smoking bans. While nurses accepted the necessity of smoking bans, they felt that there was insufficient support provided for staff to quit smoking. Acknowledgement by hospital management of the significance of tobacco addiction, provision of accessible treatment and therapy options for smokers [33], and clear communication around the smoking policy from the time of employment are important aspects of effective policy implementation.

Consistent with other studies, staff who smoked were less likely to respond positively to working in a smoke-free environment [12,19,23,27,31,34,35]. Relatively small numbers of staff were concerned about working in the smoke-free hospital before they commenced their employment. This was however higher in smokers and may be due to concerns about restrictions to their own smoking as well as perceptions of potentially increased patient aggression and loss of smoking as a management tool; views also held more strongly by staff who smoked in other studies [12,19,30]. Most staff felt that providing nicotine dependence treatment was as important as other roles in the unit, but fewer felt confident to do so. Policies should ensure provision of training and ongoing support for staff in the management of patient withdrawal and smoking cessation, within a comprehensive approach that also acknowledges and responds to staff smoking behaviour [6,16,32]. Acknowledging the impact of personal beliefs and knowledge on provision of smoking cessation support to patients is an important component of policy implementation [27]. Providing accessible cessation support to staff who smoke, and education related smoking and smoking cessation for all staff, may contribute to a culture that promotes health and enables staff to carry out their role within a smoke-free environment [26].

Just over one third of respondents believed that patients should not be forced to stop smoking, with significantly higher rates among smokers. The question of patient rights has been raised consistently as one of the barriers to implementing smoking bans in mental health inpatient facilities, along with the argument that smoking is used as self medication and that quitting will interfere with recovery [10,14,36]. While staff in mental health facilities have concerns related to rights, concerns are also held about tobacco’s highly addictive nature and it’s severe health consequences, from which disproportionately high numbers of patients with mental illness will die [10,37]. Prochaska [14] describes the perceived importance of tobacco in self medication and recovery as examples of “prevailing myths” about smoking and mental illness which are not supported by evidence [14,32]. Allowing patients to smoke during limited breaks, as a response to the issue of patient rights, adds to the regularity and persistence of nicotine withdrawal, undermines the treatment of substance abuse, and fails to provide patients with the opportunity to experience a smoke-free environment and life without smoking [10,32]. In their analysis of perceptions held by health care providers in the community mental health system, Johnson et al. [26] describe the discourses of tobacco as “therapeutic” and “an individual choice” as barriers to changing culture and practice in mental health care settings. Understanding the beliefs, culture and work environment of individual mental health care settings is an important component of policy implementation [26]. Ongoing education that challenges beliefs based on misinformation and reflects the local context may enhance staff engagement with policy implementation.

Over half of the survey respondents believed that the smoke-free environment made patient care easier. Moss et al. [10] describe the time spent by staff distributing and collecting cigarettes and lighting materials before and after each smoking break and supervising patients during the break as a negative outcome of allowing smoking in an inpatient facility. Implementation of a total smoking ban can save staff time, as provision of NRT, counseling and supporting patients takes less time than that required to supervise smoking [32,36]. Relatively small numbers of staff believed that patient behaviour had become more difficult to manage or that patients had become more aggressive. Published evaluations of smoke-free policies do not support an increase in violence and aggression following the implementation of smoking bans in mental health inpatient facilities [8,19,20,36,38]. In the small number of cases where there have been issues associated with patient aggression, reports suggest these could have been avoided with appropriate planning, and patient and staff preparation and training [33,38,39]. A total ban rather than a partial ban is more likely to be effective, providing consistency and avoiding the negative consequences of persistent nicotine withdrawal, management of smoking issues, fire risk and continued exposure to environmental tobacco smoke [4,10]. Consistent with the literature, smokers in this study were less likely to find patient care easier under a ban and were more likely to feel that there were problems with patient aggression [34,37]. As described in the literature, some staff in mental health facilities still feel that they can build rapport and develop a therapeutic relationship by smoking with patients [10,15]. While this is not a widely held view, it reflects a smoking culture that persists among some staff in mental health facilities. Alternative strategies to assess risk, manage, communicate and negotiate with patients should continue to be actively presented as more therapeutic options for staff.

The widely held perception that the smoke-free environment had a positive effect on staff and patient health provides additional support for smoke-free environments in mental health inpatient facilities. Smoking staff were less likely to agree that there had been positive health benefits, particularly in relation to their own health. Given the perceived lack of support, staff who smoke are unlikely to feel their health has improved as stress related to limitations placed on their smoking behaviour increases. Acknowledgement by management of the issues for smokers and the provision of appropriate support are aspects of policy implementation that may enhance engagement of staff who smoke.

Just under half of the survey respondents felt that mental health inpatients were unlikely to quit long term, with smoking staff significantly more pessimistic than non-smoking staff. Many people with mental illness do however want to quit and have been shown to have fairly high quit rates [2,4,14,28,32]. The impact of short term stays in non-smoking inpatient facilities on quitting is low, with the majority of patients returning to smoking on discharge [38,40]. In our follow-up (average 305 days post discharge) of a small sample of patients discharged to other mental health facilities where smoking was possible off site, 7 (58%) had remained non smokers [26]. The sample was small and the results are a likely reflection of relatively long inpatient stays experienced by the respondents. The results do however reflect the potential for long term cessation among people with severe mental illness [13,14,32]. People with a mental illness are likely to need additional support to quit long term, although paradoxically, they tend to be offered less support by health care providers [6,13,22]. Ensuring staff have the motivation, training and resources to provide information, support and access to NRT while residing in, and on discharge from, the mental health facility will be critical to long term smoking cessation by patients.

This study has a number of limitations. The return rate for the staff survey was 50% which is lower than rates reported elsewhere [17,31,34]. Staff were encouraged using different strategies over a number of weeks to complete and return the survey; the moderate return rate may indicate that the issue of smoking within the hospital is not a priority for many staff. While information on the demographics of those who did not return the survey was not available, gender and professional groupings represented by survey respondents reflected the distribution within the hospital. It is possible that non smokers may have been more likely to respond to the survey resulting in a relatively low smoking rate among staff. However given the responses of smokers to survey questions, it is likely that both smokers and non smokers felt comfortable responding to the anonymous survey and used it as an opportunity to express their views. The small number of smokers within the survey population is a limitation of the statistical analysis. The results of this study do however reflect findings of similar studies of attitudes of smokers and non-smokers [23,24]. Further, the application of these results to other mental health inpatient facilities is limited due to the unique nature of the hospital being a long stay, high secure facility. The fact that the hospital was opened as totally smoke-free facility from the outset may have influenced the make-up of the staff, with self selection of non-smoking staff on recruitment. It should be noted however that many staff (and patients) were transferred to the hospital from the correctional setting, where only partial bans applied, when the hospital opened.

## Conclusions

Important lessons from this research into a totally smoke-free policy in a high secure mental health inpatient facility, can be applied to other mental health inpatient settings. The total smoking ban was supported by the great majority of mental health professionals, with limited experience of negative outcomes in terms of patient care, behaviour and management reported. Attitudes were less positive among smokers, who perceived a lack of support in relation to their own smoking behaviour. The findings of this study highlight the importance of acknowledging the impact of individual knowledge, beliefs and smoking experience among staff. Effective policy implementation should include strategies to support staff who smoke as part of a comprehensive policy that also includes communication and ongoing training in patient smoking cessation management.

## Competing interests

The authors declare that they have no competing interests.

## Authors’ contributions

AH, DI and SP were involved in all aspects of this study from conceptualisation, development and overseeing study implementation. AH and VA implemented the surveys and interviews. AH and DI conducted the data analysis. All authors have been involved in data interpretation, and preparing the manuscript for publication. All authors read and approved the final manuscript.

## Pre-publication history

The pre-publication history for this paper can be accessed here:

http://www.biomedcentral.com/1471-2458/13/315/prepub

## References

[B1] Australian Institute of Health and Welfare (AIHW) 2010 National Drug Strategy Household Survey Report. Drug Statistics Series no. 25. Cat no. PHE 145 2011 Canberra: AIHW http://www.aihw.gov.au/publication-detail/?id=32212254712

[B2] Lasser K Wesley Boyd J Woolhandler S Smoking and mental illness. A population based prevalence study JAMA 2000 284 20 22 29

[B3] Aubin HJ Rollema H Svensson TH Winterer G Smoking, quitting and psychiatric disease: A review Neurosci Behav Rev 2012 36 1 271 284 10.1016/j.neubiorev.2011.06.007 21723317

[B4] Campion J Checinski K Nurse J McNeill A Smoking by people with mental illness and benefits of smoke free mental health services Adv Psychiatr Treat 2008 14 217 228 10.1192/apt.bp.108.005710

[B5] Ratschen E Britton J Doody GA Phil M Leonardi-Bee J McNeill A Tobacco dependence, treatment and smoke free policies: a survey of mental health professionals’ knowledge and attitudes Gen Hosp Psychiatry 2009 31 576 582 10.1016/j.genhosppsych.2009.08.003 19892217

[B6] Williams JM Steinberg ML Steinberg MB A comprehensive model for mental health tobacco recovery in New Jersey Admin Policy Ment Health 2011 38 368 383 10.1007/s10488-010-0324-x PMC3638154 21076862

[B7] Lawn S Campion J Smoke free initiatives in a psychiatric inpatient unit in a national survey of Australian sites. An Independent Report 2008 Adelaide: Flinders University

[B8] Lawn S Pols R Smoking bans in psychiatric inpatient settings. A review of the research Aust NZ J Psychiatry 2005 39 866 885 10.1080/j.1440-1614.2005.01697.x 16168014

[B9] Lawn S Campion J Factors associated with success of smoke free initiatives in Australian psychiatric inpatient units Psychiatr Serv 2010 61 3 300 305 10.1176/appi.ps.61.3.300 20194408

[B10] Moss TG Weinberger AH Vessicchia JC Mancuso V Cushing SJ Pett M Kitchen K Selby P George TP A tobacco reconceptualisation in psychiatry: toward the development of tobacco free psychiatric facilities Am J Addict 2010 19 293 311 20653636 10.1111/j.1521-0391.2010.00051.x PMC2918288

[B11] Ratschen E Britton J Doody GA Phil M McNeill A Smoke free policy in acute mental health wards: avoiding the pitfalls Gen Hosp Psychiatry 2009 31 131 136 10.1016/j.genhosppsych.2008.10.006 19269533

[B12] Dickens GL Stubbs JH Haw CM Smoking and mental health nurses: a survey of clinical staff in a psychiatric hospital J Psychiatr Ment Hlt 2004 11 445 451 10.1111/j.1365-2850.2004.00741.x 15255919

[B13] Parker C McNeill A Ratschen E Tailored tobacco dependence support for mental health patients: a model for inpatient and community services Addiction 2012 107 Suppl 2 18 25 23121356 10.1111/j.1360-0443.2012.04082.x

[B14] Prochaska JJ Smoking and mental illness – breaking the link N Engl J Med 2011 365 196 198 10.1056/NEJMp1105248 21774707 PMC4457781

[B15] Lawn S Pols R Nicotine withdrawal: pathway to aggression and assault in the locked psychiatric ward? Australas Psychiatry 2003 11 2 199 203 10.1046/j.1039-8562.2003.00548.x

[B16] Lawrence D Lawn S Kisely S Bates A Mitrou F Zubrick SR The potential impact of smoke-free facilities on smoking cessation in people with mental illness Aust N Z J Psychiatry 2011 45 1053 1060 10.3109/00048674.2011.619961 22017657

[B17] Wye P Bowman J Wiggers J Baker A Knight J Carr V Terry M Clancy R Total smoking bans in psychiatric inpatient services: a survey of perceived benefits, barriers and support among staff BMC Public Health 2010 10 372 382 10.1186/1471-2458-10-372 20576163 PMC3091547

[B18] Hollen V Ortiz G Schacht L Mojarrad M Lane GM Parks JJ Effects of adopting a smoke free policy in state psychiatric hospitals Psychiatr Serv 2010 61 9 899 904 10.1176/appi.ps.61.9.899 20810588

[B19] Voci S Bondy S Zawertailo L Walker L George TP Selby P Impact of a smoke free policy in a large psychiatric hospital on staff attitudes and patient behaviour Gen Hosp Psychiatry 2010 32 6 623 630 10.1016/j.genhosppsych.2010.08.005 21112455

[B20] Cormac I Creasey S McNeill A Ferriter M Huckstep B D’Silva K Impact of a total smoking ban in a high secure hospital The Psychiatrist 2010 34 413 417 10.1192/pb.bp.109.028571

[B21] Wye P Bowman J Wiggers J Baker A Carr V Terry M Knight J Clancy R Providing nicotine dependence treatment to psychiatric inpatients: the views of Australian nurse managers J Psychiatr Ment Hlt 2010 17 319 327 10.1111/j.1365-2850.2009.01524.x 20529182

[B22] Williams JM Steinberg ML Zimmerman MH Gandhi KK Lucas G Gonsalves DA Pearlstein I McCabe P Galazyn M Salsberg E Training Psychiatrists and Advanced Practice Nurses to treat tobacco dependence J Am Psychiatr Nurs Assoc 2009 15 50 58 10.1177/1078390308330458 21665794

[B23] Parks T Wilson CVR Turner K Chin JWE Failure of hospital employees to comply with smoke-free policy is associated with nicotine dependence and motives for smoking: a descriptive cross-sectional study at a teaching hospital in the United Kingdom BMC Public Health 2009 9 238 10.1186/1471-2458-9-238 19604348 PMC2720965

[B24] Ravara SB Calheiros JM Aguiar P Barata LT Smoking behaviour predicts tobacco control attitudes in a high smoking prevalence hospital: A cross-sectional study in a Portuguese teaching hospital prior to the national smoking ban BMC Public Health 2011 11 720 10.1186/1471-2458-11-720 21943400 PMC3189890

[B25] Slater P McElwee G Fleming P McKenna H Nurses’ smoking behaviour related to cessation practice Nurs Times 2006 102 95 32 37 16711288

[B26] Johnson JL Moffat BM Malchy LA In the shadow of a new smoke free policy: A discourse analysis of health care providers’ engagement in tobacco control in community mental health Int J Ment Health Syst 2010 4 23 34 10.1186/1752-4458-4-23 20667105 PMC2920253

[B27] Knudsen HK Studts JL The implementation of tobacco-related brief interventions in substance abuse treatment: A national study of counselors J Subst Abuse Treat 2010 38 212 219 10.1016/j.jsat.2009.12.002 20116960 PMC2864720

[B28] Hehir A Indig D Prosser S Archer V Evaluation of a smoke-free forensic mental health inpatient hospital: the patient perspective Drug Alcohol Rev 2012 31 672 677 10.1111/j.1465-3362.2012.00456.x 22524262

[B29] NSW Department of Health NSW Health Smoke-Free Workplace Policy 1999 Sydney

[B30] Stubbs J Haw C Garner L Survey of staff attitudes to smoking in a large psychiatric hospital The Psychiatrist 2004 28 204 207

[B31] Bloor RN Meeson L Crome IB The effects of a non smoking policy on nursing staff smoking behaviour and attitudes in a psychiatric hospital J Psychiatr Ment Hlt 2006 13 188 196 10.1111/j.1365-2850.2006.00940.x 16608474

[B32] Prochaska JJ Ten critical reasons for treating tobacco dependence in inpatient psychiatry J Am Psychiatr Nurses Assoc 2009 15 404 409 10.1177/1078390309355318 20336177 PMC2844659

[B33] Hempel AG Kownacki R Malin DH Ozone SJ Cormack TS Sandoval BG Leinbach AE Effect of a total smoking ban in a maximum security psychiatric hospital Behav Sci Law 2002 20 507 522 10.1002/bsl.503 12239709

[B34] Garg S Shenoy S Badee M Varghese J Quinn P Kent J Survey of staff attitudes to the smoking ban in a medium secure unit J Forensic Leg Med 2009 16 378 380 10.1016/j.jflm.2009.04.009 19733324

[B35] Dart GS van Beurden EK Zask A Lord C Kia AM Tokley R Reduction in staff smoking rates in North Coast Area Health Service, NSW, following the introduction of a smoke-free workplace policy NSW Public Health Bull 2010 21 12 263 266 10.1071/NB10036 21426852

[B36] Williams JM Eliminating tobacco use in mental health facilities JAMA 2008 299 5 571 573 10.1001/jama.299.5.571 18252888

[B37] Dwyer T Bradshaw J Happell B Comparison of mental health nurses’ attitudes towards smoking and smoking behaviour Int J Ment Health Nurs 2009 18 424 433 10.1111/j.1447-0349.2009.00628.x 19883414

[B38] Shetty A Alex R The experience of a smoke free policy in a medium secure hospital The Psychiatrist 2010 34 287 289 10.1192/pb.bp.109.027425

[B39] Campion J Lawn S Brownlie A Hunter E Gynther B Pols R Implementing smoke-free policies in mental health inpatient units: learning from unsuccessful experience Australas Psychiatry 2008 16 2 92 97 10.1080/10398560701851976 18335364

[B40] Prochaska JJ Fletcher L Hall SE Hall SM Return to smoking following a smoke free psychiatric hospitalization Am J Addict 2006 15 15 22 10.1080/10550490500419011 16449089

